# The Activity of Alcohol Dehydrogenase (ADH) Isoenzymes and Aldehyde Dehydrogenase (ALDH) in the Sera of Patients with Brain Cancer

**DOI:** 10.1007/s11064-014-1402-3

**Published:** 2014-10-10

**Authors:** Wojciech Jelski, Magdalena Laniewska-Dunaj, Karolina Orywal, Jan Kochanowicz, Robert Rutkowski, Maciej Szmitkowski

**Affiliations:** 1 Department of Biochemical Diagnostics, Medical University, Waszyngtona 15 A, 15-269 Bialystok, Poland; 2 Department of Neurosurgery, Medical University, Bialystok, Poland

**Keywords:** Alcohol dehydrogenase isoenzymes, Aldehyde dehydrogenase, Brain cancer

## Abstract

Human brain tissue contains various alcohol dehydrogenase (ADH) isoenzymes and possess also aldehyde dehydrogenase (ALDH) activity. In our last experiments we have shown that ADH and ALDH are present also in the brain tumour cells. Moreover the activities of total ADH and class I isoenzymes were significantly higher in cancer tissue than healthy cells. It can suggests that these changes may be reflected by enzyme activity in the serum of patients with brain cancer. Serum samples were taken for routine biochemical investigation from 62 patients suffering from brain cancer (36 glioblastoma, 26 meningioma). For the measurement of the activity of class I and II ADH isoenzymes and ALDH activity, the fluorometric methods were used. The total ADH activity and activity of class III and IV isoenzymes were measured by the photometric method. A statistically significant increase of class I alcohol dehydrogenase isoenzymes was found in the sera of patients with brain cancer. The median activity of this class isoenzyme in the patients group increased about 24 % in the comparison to the control level. The total alcohol dehydrogenase activity was also significantly higher (26 %) among patients with brain tumour than healthy ones. The activities of other tested ADH isoenzymes and total ALDH were unchanged. The increase of the activity of total ADH and class I alcohol dehydrogenase isoenzyme in the sera of patients with brain cancer seems to be caused by the release of this isoenzyme from tumour’s cells.

## Introduction

Malignant tumours of the brain are accounting for approximately 2 % of all cancers in adults. Approximately 4,400 people are newly diagnosed with a brain tumour each year in the developed countries. Primary brain cancers have attracted increased attention in recent decades because, although the survival rate continues to remain poor, there are several reports suggesting an increasing trend in incidence rates [1]. It is characterized by relatively late stage of diagnosis. Therefore it is important to find markers that may be helpful for diagnosing of brain cancer. The best solution for the diagnosis of patients with central nervous system (CNS) tumours could be easily available biomarkers, which could be useful for the management of CNS neoplasms.

Some investigations have shown that alcohol dehydrogenase (ADH) and aldehyde dehydrogenase (ALDH) are present in the cells of human brain tissues [2, 3]. ADH and ALDH, the enzymes responsible for metabolism of many biological substances such as ethanol, serotonin, retinol, exist in multiple molecular forms that have been grouped into several classes, which display tissue specificity [4]. In previous study we have shown that ADH and its isoenzymes and ALDH are present in the brain cancer cells. Moreover the total activities of alcohol dehydrogenase and class I ADH isoenzymes were significantly higher in cancer tissues than in healthy brain cells [5]. We hypothesized that changed activities of these enzymes in the cancer cells can play role in carcinogenesis and would be reflected in the serum. In this study we investigated the activity of alcohol dehydrogenase and its isoenzymes and the total activity of ALDH in the sera of patients with brain cancer.

## Materials and Methods

### Patients

The protocol was approved by the Human Care Committee of the Medical University in Bialystok, Poland (Approval Nr R-I-002/460/2011). All patients gave informed consent for the examination.

Serum samples were taken for routine biochemical investigations from 62 patients suffering from brain tumours (35 males and 27 females, range 30–77 years). All tumors patients were identified from January 2011–December 2012 in Department of Neurosurgery Medical University of Bialystok (Poland). Among the cancer patients, 36 patients (21 men and 15 women) suffering from glioblastoma and 26 patients (14 men, 12 women) had meningioma. The tumors were histopathologically classified according to World Health Organization criteria. We have created the following groups, depending on the location of the tumor: the frontal lobe, temporal lobe, parietal lobe, cerebellum. Classification was based on preoperative computed tomography (CT) and magnetic resonance imaging scans. Preoperative functional status of the patient was evaluated with the Karnofsky Performance Status Scale. Preoperative MRI was used to determine tumor volumes. None of the patients had received chemotherapy or radiotherapy before samples collection. All of the patients drank alcohol occasionally and self-reported an intake of <25 g of ethanol per week. The clinicopathological characteristics of the patients are shown in Table 1.

**Table 1:** Characteristics of brain cancer patients

Variable	No of patients
Brain cancer patients	62
Gender	
Males	37
Females	27
Age	
<50 years	34
≥50 years	28
Range	30–77
Histological type	
Glioblastoma	36
Meningioma	26
Tumour stage	
I	16
II	10
III	15
IV	21
Tumor size	
<3 cm	35
≥3 cm	27
Location in the brain	
Frontal lobe	16
Temporal lobe	24
Parietal lobe	16
Cerebellum	6
Degree of differentiation	
Well	39
Poorly	23

Serum samples were also obtained from 120 healthy subjects (control group, 70 males, 50 females, aged 35–65 years). Control groups were selected from healthy community residents who attend the hospitals for routine physical check-ups at the Department of Preventive Medicine. One and all were volunteers and were defined as those with normal results of all physical, blood examinations and CT of the brain. Before the examinations control group had not consumed alcohol almost 1 months and ethanol did not exist in serum samples of any subject when it was collected.

### Biochemical Assays

#### Determination of Total ADH Activity

Total ADH activity was estimated by the photometric method with p-nitrosodimethylaniline (NDMA) as a substrate [6]. The reaction mixture (2 ml) contained 0.1 ml of serum and 1.8 ml of a 26 μM solution of substrate in 0.1 M of sodium phosphate buffer, pH 8.5 and 0.1 ml of a mixture containing 0.25 M n-butanol and 5 mM NAD. The reduction of NDMA was monitored at 440 nm on a Shimadzu UV/VIS 1202 spectrophotometer (Shimadzu Europa GmbH, Duisburg, Germany).

#### Determination of Total ALDH Activity

Aldehyde dehydrogenase activity was measured using the fluorogenic method based on the oxidation of 6-methoxy-2-naphtaldehyde to the fluorescent 6-methoxy-2 naphtoate [7]. The reaction mixture contained 60 μl of serum, 60 μl of substrate, 20 μl of 11.4 mM NAD and 2.8 ml of 50 mM of sodium phosphate buffer, pH 8.5. The mixture contained also 50 μl of a 12 mM solution of 4-methylpyrazole as a specific inhibitor of ADH activity. The fluorescence was read at excitation wavelength 310 and emission wavelength 360 nm.

#### Determination of Class I and II ADH Isoenzymes

Class I and II alcohol dehydrogenase isoenzyme activity were measured using fluorogenic substrates (4-methoxy-1-naphthaldehyde for class I and 6-methoxy-2-naphthaldehyde for class II) in reduction reaction according to Wierzchowski [8]. The assays were performed in a reaction mixture containing a serum (60 μl), substrate (150 μl of 300 μM), NADH (100 μl of 1 mM) and 0, 1 M of sodium phosphate buffer, pH 7.6 (2.69 ml) in conditions previously described [9]. The measurements were performed on a Shimadzu RF–5301 spectrofluorophotometer (Shimadzu Europa GmbH, Duisburg, Germany) at excitation wavelength 316 nm for both substrates and emission of 370 nm for class I and 360 nm for class II isoenzymes.

#### Determination of Class III ADH Isoenzyme

The assay mixture for class III of alcohol dehydrogenase activity contained a serum (100 μl), n–octanol as a substrate (31 μl of 1 mM), NAD (240 μl of 1.2 mM) in 0.1 M NaOH-glycine buffer pH of 9, 6 [10]. The reduction of NAD was monitored at 340 nm and 25 °C on a Shimadzu UV/VIS 1202 spectrophotometer.

#### Determination of Class IV ADH Isoenzyme

The assay mixture for class IV of alcohol dehydrogenase activity contained a serum (50 μl), m–nitrobenzaldehyde as a substrate of (132 μl of 80 μM), NADH (172 μl of 86 μM) in 0.1 M sodium phosphate buffer pH 7.5 [11]. The oxidation of NADH was monitored at 340 nm and 25 °C on a Shimadzu UV/VIS 1202 spectrophotometer.

### Statistical Analysis

A preliminary statistical analysis (Chi square test) revealed that the distribution of ADH and ALDH activities did not follow a normal distribution. Consequently, the Wilcoxon’s test was used for statistical analysis. Data were presented as median, range and mean values. Statistically significant differences were defined as comparisons resulting in *p* < 0.05.

## Results

The activities of alcohol dehydrogenase, aldehyde dehydrogenase and isoenzymes of alcohol dehydrogenase in the sera are demonstrated in Table 2. The total activity of alcohol dehydrogenase was significantly higher (about 26 %) in the serum of patients with brain cancer than in healthy subjects. The median total activity of ADH was 895 mU/l in patients group and 664 mU/l in control group. The analysis of ALDH activity did not indicated significant difference between total tested group and healthy controls. The comparison of ADH isoenzymes activities showed that the highest difference was exhibited by class I ADH. The median activity of this class isoenzyme in the total cancer group increased about 24 % (1.77 mU/l) in comparison to the control level (1.35 mU/l). This increase was statistically significant. The other tested classes of ADH isoenzymes had higher activities in the serum of patients with brain cancer, but the differences were not statistically significant in all patients groups (*p* > 0.05).

**Table 2:** ADH and ALDH activity in the sera of patients with brain tumour

Tested group	ADH I	ADH II	ADH III	ADH IV	ADH Total	ALDH
	Median	Median	Median	Median	Median	Median
	Range	Range	Range	Range	Range	Range
	Range	Mean	Mean	Mean	Mean	Mean
Brain cancer (n = 62)	1.77	14.81	12.23	5.46	895	2.90
	1.08–3.64	6.94–21.65	7.05–18.21	2.11–11.98	338–1,544	1.35–5.63
	1.73	14.24	12.16	5.31	866	2.77
Glioblastoma (n = 36)	1.79	14.96	12.55	5.64	924	3.02
	1.11–3.64	7.25–21.65	7.62–18.21	2.36–11.98	366–1,544	1.48–5.63
	1.76	14.55	12.33	5.40	901	2.88
Meningioma (n = 26)	1.73	14.52	12.14	5.32	880	2.84
	1.08–3.32	7.25–20.48	7.05–18.03	2.11–11.56	338–1,488	1.35–5.14
	1.71	14.18	12.05	5.21	856	2.68
Control (n = 120)	1.35	14.45	11.96	5.22	664	2.74
	0.96–2.83	6.06–19.82	7.22–17.48	2.04–11.75	267–1,345	1.21–5.36
	1.31	14.02	11.35	5.08	740	2.58
	p^a^ < 0.001*	p^a^ = 0.327	p^a^ = 0.296	p^a^ = 0.436	p^a^ < 0.001*	p^a^ = 0.274
	p^b^ < 0.001*	p^b^ = 0.366	p^b^ = 0.346	p^b^ = 0.372	p^b^ < 0.001*	p^b^ = 0.369
	p^c^ < 0.001*	p^c^ = 0.258	p^c^ = 0.377	p^c^ = 0.348	p^c^ < 0.001*	p^c^ = 0.361
	p^d^ = 0.437	p^d^ = 0.436	p^d^ = 0.281	p^d^ = 0.274	p^d^ = 0.423	p^d^ = 0.285

Data are expressed as mU/l

*p* ^*a*^ brain cancer versus control

*p* ^*b*^ glioblastoma versus control

*p* ^*c*^ meningioma versus control

*p* ^*d*^ glioblastoma versus meningioma

* statistically significant differences between suitable groups

The analysis of ADH, ALDH and ADH isoenzymes activities in the serum did not indicate significant differences between patients with glioblastoma and meningioma.

The analysis of ADH, ALDH and ADH isoenzymes activities showed lack of statistically significant difference depending on the location of the tumor (Table 3).

**Table 3:** The comparison of ADH isoenzymes and ALDH activity in the serum of patients with brain cancer depending on the localization of tumour

Tested group	ADH I	ADH II	ADH II	ADH IV	ADH Total	ALDH
	Median	Median	Median	Median	Median	Median
	Range	Range	Range	Range	Range	Range
	Mean	Mean	Mean	Mean	Mean	Mean
Frontal lobe (n = 16)	1.82	14.85	12.42	5.51	906	2.98
	1.21–3.47	7.24–21.65	7.34–18.04	2.18–11.77	378–1,544	1.48–5.63
	1.71	14.47	12.27	5.40	895	2.84
Temporal lobe (n = 24)	1.76	14.58	12.17	5.42	881	2.85
	1.15–3.36	7.03–21.14	7.16–17.86	2.23–11.58	361–1,492	1.48–5.28
	1.68	14.22	12.09	5.28	870	2.74
Parietal lobe (n = 16)	1.70	14.55	12.11	5.35	876	2.87
	1.08–3.03	6.94–20.95	7.05–17.67	2.11–11.61	338–1,465	1.35–5.22
	1.62	14.31	12.03	5.16	862	2.70
Cerebellum (n = 6)	1.88	14.76	12.47	5.48	899	2.94
	1.31–3.64	7.12–21.30	7.46–18.21	2.28–11.98	353–1,518	1.52–5.47
	1.80	14.38	12.30	5.36	883	2.88
Control (n = 120)	1.35	14.45	11.96	5.22	664	2.74
	0.96–2.83	6.06–19.82	7.22–17.48	2.04–11.75	267–1,345	1.21–5.36
	1.31	14.02	11.35	5.08	740	2.58

Data are expressed as mU/l

## Discussion

Brain tumours in adults are a rare diseases from which survival is generally poor compared to many other cancers. However survival chances have improved gradually over the last 30 years. The length of survival following diagnosis of a brain tumour is dependent on the age of the patient, histologic subtype, grade of the tumour, and presenting symptoms [12]. Therefore it is very important to find markers which would detect a malignant cell transformation as early as it is possible. Tumour markers should be synthesized and excreted by tumour tissues [13]. Currently, except for rare germ cell tumors, there is a lack of knowledge on biochemical markers for CNS neoplasms.

The data that have been published concerning the localization and activity of different ADH classes in the brain are partially contradictory. Some researchers demonstrated that only ADH III is present in the brain [14, 15]. Expression of ADH I and ADH IV mRNA in the adult brain has been claimed by study using partially hydrolyzed riboprobe in situ hybridization. The authors found ADH I and ADH IV in cerebellar granule cells and different regions of cerebral cortex [4]. Thus human brain express mainly isoenzymes of three classes of alcohol dehydrogenase: I, III, and IV. In our previous study we have found that alcohol dehydrogenase isoenzymes and aldehyde dehydrogenase are present in the brain cancer cells. Moreover we also shown that the total activity of ADH in cancer tissue is about 28 % higher than that in healthy brain tissues and there is no statistical differences in the activity of aldehyde dehydrogenase between brain healthy and cancerous tissues. In addition the data of the same study showed that only the activity of class I ADH is significantly higher in cancer than in healthy brain cells. ADH I is the main ethanol-metabolizing isoenzyme class in the brain. The other tested classes of ADH isoenzymes had higher activities in cancer tissue than in healthy brain cells, but these differences were not statistically significant [5]. In present study we stated that the serum total alcohol dehydrogenase activity has been significantly increased in brain cancer. The significant increase of total activity of ADH was positively correlated with ADH I so it indicates that the cause of the significant increase of serum total alcohol dehydrogenase in the course of brain cancer is an elevation of class I ADH isoenzymes. The increases of other ADH isoenzymes activities were not significant in the serum of patients with brain cancer. In our opinion low activity of ADH II, III and IV can be explained by low activity of these isoenzymes in the brain cancer cells and by low secretion into the sera. Aldehyde dehydrogenase is present in the brain although the activity of ALDH seems to be disproportionaly low to ADH activity. The serum levels of aldehyde dehydrogenase were not significantly higher in patients with brain tumour in comparison to healthy group.

The analysis of ADH, ALDH and ADH isoenzymes activities in the serum did not indicate significant differences between patients with glioblastoma and meningioma. These data are in accordance with our previous study. The histological differences of cancers did not effect the activity of tested enzymes. We have found that the activity of ADH and ALDH in cancer tissues of glioblastoma did not differ from that of meningioma. It is surprising, because they differ in the degree of malignancy, the origin of the cells and etiology.

Alcohol dehydrogenase I activity was found in the cerebellar granule cells and Purkinje cells, in the hippocampal formation, cerebellum and other regions of cerebral cortex [4]. By Western blot analysis and immunohistochemistry, highest ADH III levels were found in cerebellum and hippocampus and lower concentrations in other parts of cortex cerebri [16]. In this study, we examined the dependence of the ADH activity in serum and localization of brain tumor. Analyzing the activity of ADH, ALDH and ADH isoenzymes we do not find the significant differences depending on the location of the tumor in the brain.

In our previous investigations we have presented data that the activity of class I ADH (the principal class of ADH isoenzymes in the liver and colon is significantly higher in cancer cells than in healthy liver or colorectum [17, 18]. Therefore, the activity of class I alcohol dehydrogenase isoenzymes was significantly elevated in the serum of patients with liver and colorectal cancer [19, 20].

In conclusion, we can state that the increase of the activity of total ADH and class I alcohol dehydrogenase isoenzyme in the sera of brain cancer patients seems to be caused by release of this isoenzyme from cancer cells to blood and probably total ADH and ADH I determination in serum could be helpful for diagnostic of CNS tumours but further investigations and confirmation by a prospective study are necessary.
